# Integrative Transcriptomic Analysis Identify Potential m6A Pathway-Related Drugs That Inhibit Cancer Cell Proliferation

**DOI:** 10.3390/genes13112011

**Published:** 2022-11-02

**Authors:** Jingkun Yi, Rucong Liu, Yu Liu, Ting Guo, Yang Li, Yuan Zhou

**Affiliations:** 1 Department of Biomedical Informatics, MOE Key Lab of Cardiovascular Sciences, School of Basic Medical Sciences, Peking University, Beijing 100191, China; 2 Department of Cell Biology, School of Basic Medical Sciences, Peking University Stem Cell Research Center, Peking University, Beijing 100191, China; 3 Department of Gastrointestinal Translational Research, Key Laboratory of Carcinogenesis and Translational Research (Ministry of Education), Peking University Cancer Hospital & Institute, Beijing 100021, China

**Keywords:** m6A methylation, transcriptome, connectivity map, cancer cell proliferation, drug

## Abstract

Recent studies have found that m6A modification of mRNA may play important roles in the progression of various types of cancers. However, current knowledge about drugs that can interfere with m6A methylation and inhibit cancer cell proliferation is still far from comprehensive. To this end, we performed integrative analysis on transcriptome data with perturbation of m6A writers or erasers and identified consensus m6A-related differentially expressed genes (DEGs). Comparative analysis of these m6A-related DEGs with Connectivity Map signatures highlight potential m6A-targeted drugs. Among them, we experimentally verified the inhibitory effects of AZ628 on the proliferation of human breast cancer cell lines and R428 on the proliferation of human melanoma cell lines. Both drugs can significantly reduce the cellular level of m6A modification. These results suggest an m6A-related new target pathway by AZ628 and R428 and provide new candidate m6A-related drugs that inhibit cancer cell proliferation.

## 1. Introduction

Recent studies reveal that various types of mRNA modifications contribute to the regulations and functions of mRNAs; among these mRNA modifications, m6A methylation modification is the most common type [1]. There are three categories of proteins that directly affect the biological effects of m6A modification: writers, erasers, and readers [2]. Briefly, writers (METTL3, METTL14 and others) add m6A modifications to mRNAs, erasers (FTO and ALKBH5) remove m6A modifications, and readers (YTH family protein and others) guide downstream functional readouts [2]. Together, these three enzymes constitute a dynamic m6A-centric regulatory system. This regulatory system plays important roles in a variety of cellular processes. For example, m6A methylation can regulate the proliferation and differentiation of stem cells. When m6A is knocked down, stem cells will lose the ability to self-renewal [3], which suggests that m6A modification is an essential step in normal cell proliferation.

Related to its roles in cell proliferation control, m6A modification also plays an important role in the initiation and progression of a wide range of cancer types [4,5,6,7,8,9]. Divergence impacts of m6A modification were observed in different types of cancers. For example, the m6A modified writer *METTL3* exists as an oncogenic gene in some cancer types such as leukemia, breast cancer and liver cancer [10,11,12] but is a tumor suppressor gene in other cancer types such as glioblastoma and endometrial cancer [13,14]. These different effects may be attributed to the fact that METTL3 mainly acts on different genes in different cancers, and the addition of m6A modification to different genes ultimately leads to different biological effects, thus showing conflicting effects in different cancers. Thus, an integrative analysis of the multiple m6A-related transcriptome data is substantial to obtain a robust m6A-related transcriptomic signature which is helpful for further predictive analyses like drug prediction.

Indeed, recent studies have revealed new anti-cancer drugs that target m6A pathways. For example, m6A eraser FTO is the target of many anticancer drugs. The R enantiomer of 2-hydroxyglutarate (R-2HG) is a broad-spectrum inhibitor that can exert anti-leukemic effects by promoting cell cycle arrest and apoptosis. Studies have found that R-2HG can inhibit activity of the m6A eraser FTO, increase m6A modification, and reduce the stability of the MYC/CEBPA transcript to exert an anti-cancer effect [15]. Huang et al. designed the compound FB23-2 based on the FTO protein structure. The FB23-2 compound not only showed specific inhibition against FTO and proliferation against the AML cancer cell line in vitro, but also inhibited the progression of leukemia and prolonged the survival time in an in vivo AML model [16]. In addition to FTO, the m6A writer METTL3 has also become a new target for anti-cancer drug development. As a selective METTL3 inhibitor, STM2457 was found to reduce the growth and increase the differentiation and apoptosis of AML; these effects on cells are accompanied by a decrease in the amount of m6A modification [17]. Nevertheless, current knowledge about anti-cancer drugs through targeting the m6A methylation pathway is still far from comprehensive. Therefore, in this study, we first collected transcriptome data with gene knockdown or overexpression of the m6A writers and erasers and identified consensus up and down of differentially expressed genes (DEGs) that correlate with the increasing and decreasing m6A levels, respectively. Connectivity Map (CMap) analysis was conducted according to these consensus DEGs to find the drugs that might be effective and interfere with m6A methylation and inhibit cancer cell proliferation. Finally, we conducted experiment validations on the corresponding drugs to check the cell proliferation curve, the protein level of the m6A writer METTL3 and the total amount of m6A modification after the treatment of these drugs.

## 2. Materials and Methods

### 2.1. Collection and Processing of m6A-Related Transcriptome Datasets

The m6A-related transcriptome datasets included in this study were obtained from Gene Expression Omnibus (GEO). More specifically, we selected the m6A writer knockdown (KD) study GSE81164, GSE147884, GSE161301, GSE161302, GSE182382 and GSE190078; the m6A writer knockout (KO) study GSE95372, GSE130012; the m6A writer overexpression (OE) study GSE179267, GSE186581, the m6A eraser KD study GSE103497, GSE107411, GSE128574, GSE133517, GSE144959, GSE144968, GSE146874 and GSE182382. Among them, the datasets GSE95372, GSE144959, GSE144968, GSE161302 and GSE179267 in the database do not include raw counts data for further differential gene expression analysis. Therefore, we performed a transcriptome data analysis pipeline to obtain the raw count expression profiles of these datasets. Briefly, the SRA-Toolkit (V3.0.0) was used to fetch the FASTQ reads, the fastp (V0.21.1) was used to remove the low-quality connectors, and the STAR (V2.7.8a) was used to map FASTQ reads to the human genome. Finally, RSEM (V1.3.1) was used to calculate the raw count expressions. After all raw count expression profiles were obtained, the makeContrasts function of the limma package (V3.50.3) [18] of R (V4.0.3) was used for the differential gene expression analysis. Notably, for the group with reduced m6A modification (including those with writer KO/KD), the differentially expressed genes were calculated by the control group versus the experimental group, while for the group with increased m6A modification (including those with eraser KO/KD and writer OE), the differentially expressed genes were calculated by the experimental group versus the control group. The direction of DEGs were in line with the direction of the m6A level changes in the experiments.

### 2.2. Identification and Functional Enrichment Analysis of Consensus m6A-Related Differentially Expressed Gene (DEG)

Before the identification of consensus DEGs, we first compared the internal correlation of different data, and excluded six studies (GSE81164, GSE128574, GSE144968, GSE161301, GSE161302, and GSE179267) with prominent differences of DEGs in compared to other datasets. After removing these six studies, we performed a meta-analysis on the remaining 12 studies using the MetaVolcanoR package (V1.8.0) in R and obtained the consensus up-regulated and down-regulated genes. Genes showing |Foldchange| > 1.5 were retained. We used the Clusterprofiler package (V4.2.2) [19] and the ReactomePA package (V1.38.0) in R for Gene Ontology (GO) enrichment analysis and Reactome pathway analysis of those genes. When Padj is less than 0.05, the GO term and Reactome pathway analysis was considered significantly enriched among these genes.

### 2.3. Prediciton of Potential m6A Methylation-Tagreted Durgs by Connectivity Map (CMap) Analysis

Because CMap analysis often requires a small and high confident set of DEGs as its input, we further filtered the consensus DEGs by restricting up-regulated genes with signcon > 1, het_QMp < 0.05 and Fold-change > 1.5 and down-regulated genes with signcon < −1, het_QMp < 0.05 and Fold-change < −1.5. SigCom-LINCS (https://maayanlab.cloud/sigcom-lincs#/SignatureSearch/UpDown, accessed on 10 June 2021) which was used to predict the potential drugs based on the comparative analysis with LINCS CMap profiles. For the predicted drugs obtained by this analysis, we only retained those from cancer cell lines that were treated with a dose of less than 2 μmol to further prioritize the potential anti-cancer drugs.

### 2.4. Cell Culture and Cell Proliferation Assay

The breast cancer cell line MCF7 and the human melanoma cell line A375 were provided by Biomol Inc. (Hamburg, Germany). The MCF7 and A375 cells were cultured in DMEM medium (Gibco, Carlsbad, CA, USA) containing 10% fetal bovine serum (FBS, Gemini, CA, USA) and 1% penicillin-streptomycin (Gibco, Carlsbad, CA, USA). Then A375 (2.0 × 10^6^) and MCF7 (1.0 × 10^6^) cells were inoculated into 60 mm cell culture dishes. The A375 cells were divided into two groups: the control group was treated with the dimethyl sulfoxide (DMSO) solvent (1.11 μmol, Solarbio, Beijing, China) for 24 h, and the experimental group was treated with the DMSO-dissolved R-428 solution (1.11 μmol, MedChemExpress, Monmouth Junction, NJ, USA) for 24 h. The MCF7 cells were also divided into two groups: the control group was treated with the DMSO solvent (1.11 μmol, Solarbio, Beijing, China) for 24 h and the experimental group was treated with the DMSO-dissolved AZ628 solution (1.11 μmol, MedChemExpress, Monmouth Junction, NJ, USA) for 6 h. After treatment, single-cell suspensions were digested with trypsin (Gibco, Carlsbad, CA, USA) every 24 h, and trypan blue (Beyotime, Shanghai, China) was added to exclude dead cells, and cells were subsequently counted in counting plates. The number of cells counted in the cell counting plate was used to calculate the total number of cells in the dish, and the cell proliferation curve was made with time as the horizontal axis and the total number of cells as the vertical axis.

In addition, the effects of drugs on the proliferation of the A375 and MCF7 cells were also verified by the MTT assay. Cells were seeded into 96-well plates at a density of 3000 cells per well. Cell viability was measured at 0, 24, 48, 72, and 96 h after inoculation using the MTT cell proliferation and cytotoxicity assay kit (Leagene Biotechnology, Beijing, China). First, 10 μmol of MTT solution and 100 μmol of complete DMEM medium were added to each well, and then incubated at 37 °C and 5% CO_2_ for 4 h. Finally, 110 μmol of Formazan solvent was added to each well and shaken for 10 min. The absorbance was measured by microplate analyzer at 570 nm.

### 2.5. Western Blot

RIPA lysis buffer (Beyotime, Shanghai, China) was used to lyse the cells on ice which were then centrifuged 12,000× *g* at 4 °C for 15 min. The BCA protein determination kit (Beyotime, Shanghai, China) was used to determine the protein content. The proteins were isolated by 10% SDS-PAGE and transferred to PVDF membranes (Merck-Millipore, Darmstadt, Germany). Membranes were blocked with 5% skimmed milk (Beyotime, Shanghai, China) for 1 h and probed overnight with anti-METTL3 antibody (1:1000, Cat.#E3F2A, Cell Signaling Technology, Danvers, MA, USA) and anti-GAPDH antibody (1:1000, Cat.#AC001, Cell Signaling Technology, Danvers, MA, USA) at 4 °C overnight, and followed by incubation with HRP-conjugated secondary anti- IgG antibody (1:1000, Beyotime, Shanghai, China) for 1 h at room temperature. Membranes were detected with HRP substrate luminol reagents (Merck-Millipore, Darmstadt, Germany), and scanned using the Gel Doc EZ Imager (Bio-Rad, Berkeley, CA, USA). Quantification of blot intensity was performed using ImageJ software (V1.8.0, NIH, MD).

### 2.6. m6A RNA Methylation Quantification

RNA was first extracted from cells using TRIzol (Invitrogen, Carlsbad, CA, USA) and then isolated and obtained using trichloromethane and isopropanol. The EpiQuik^TM^ m6A RNA Methylation Quantification Kit (Colorimetric) (Epigentek, Farmingdale, NY, USA) was used to test the total amount of m6A modification. The standard protocol was applied as per the manufacturer’s guidelines. First, 80 μL of binding solution and 200 ng RNA solution were added to the 96-well plate, and the plates were incubated at 37 °C for 90 min. After incubation, the binding solution was discarded and washed three times with wash buffer. Each well was treated with 50 μL of Capture Antibody for 60 min. Capture Antibody was abandoned and washed three times with wash buffer. Each well was treated with 50 μL of Detection Antibody for 30 min. The Detection Antibody was abandoned and washed four times with wash buffer and 50 μL of Enhancer Solution was added to each well for 30 min. The Enhancer Solution was discarded and washed with wash buffer five times. Then, 100 μL of Developer Solution was added and incubated in the dark for 10 min. Next, 100 μL of Stop Solution was added and the absorbance was measured at 450 nm. The concentration curve was drawn and the corresponding m6A modification was calculated.

## 3. Results

### 3.1. Identification of Consensus DEGs from Multiple Transcriptome Datasets with m6A Writer or Eraser Pertubations

We first searched the datasets with the perturbation of m6A modifying enzyme genes in the GEO database, mainly focusing on four genes: m6A writer *METTL3*, *METTL14*, m6A eraser *FTO* and *ALKBH5*. The gene expression data related to knockdown (KD), knockout (KO) and overexpression of these genes was collected and processed if no raw count expression matrix was provided. The specific datasets we selected are summarized in Table 1.

**Figure 1:**
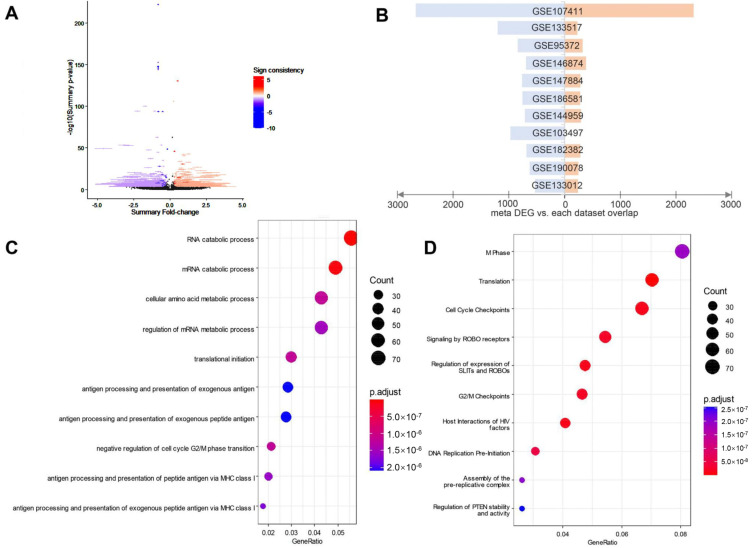
Overview of the m6A-related consensus differentially expressed genes (DEGs). (**A**) Volcano plot of meta-analysis. (**B**) Coincidence number of DEGs in individual datasets. (**C**) GO enrichment analysis of the consensus DEGs from meta-analysis. (**D**) Reactome pathway analysis of the consensus DEGs from meta-analysis.

To find the consensus DEG signature, the direction of differential expression should first be unified. To this end, we divided the datasets into two groups according to the increase or decrease of the m6A level after perturbation. More specifically, the datasets regarding the m6A writer KD/KO were taken as the m6A decrease group, while the datasets of m6A eraser KD and m6A writer overexpression were assigned as the m6A increase group. For the m6A decrease group, we used the control group versus the experimental group to conduct differential gene analysis. And for the m6A increase group, we used the experimental group versus the control group to conduct differential gene analysis. By this configuration, the up-and down-regulation direction of the DEGs should line with the up- and down-regulation of corresponding m6A levels. After removing datasets with highly diverged DEG sets, 6626 consensus up-regulated DEGs and 12,649 consensus down-regulated DEGs were obtained. We compared the consensus DEGs from the meta-analysis with the up-regulated and down-regulated genes from the specific dataset to evaluate their similarity and found that there is a certain amount of overlap between most of the data sets and the meta-analysis results, which indicate that the meta-analysis results are at least partly related to the individual result from each dataset (Figure 1B).

### 3.2. Enriched Functions of the m6A-Related Consensus DEGs

We conducted a GO functional enrichment analysis and a Reactome pathway analysis on these consensus DEGs. The results of the GO enrichment analysis show that the consensus DEGs obtained from the meta-analysis are related to the regulation of mRNA metabolic process RNA and RNA catabolic process (Figure 1C). Reactome pathway analysis show that DEGs were significantly related to mitotic phase, translation, cell cycle checkpoints and ROBO receptor pathways (Figure 1D), which are essential for cell proliferation. There is no doubt that mitosis and translation processes are highly activated in cancer cells; the ROBO receptor pathway is also found to be activated in some cancers [20], and cell cycle checkpoints are prone to appear abnormal in cancer cells [21]. The results obtained from DEG analysis is closely related to cancer cell division and proliferation.

### 3.3. Prediction of the Potential m6A-Realted Anti-Cancer Drugs

**Figure 2:**
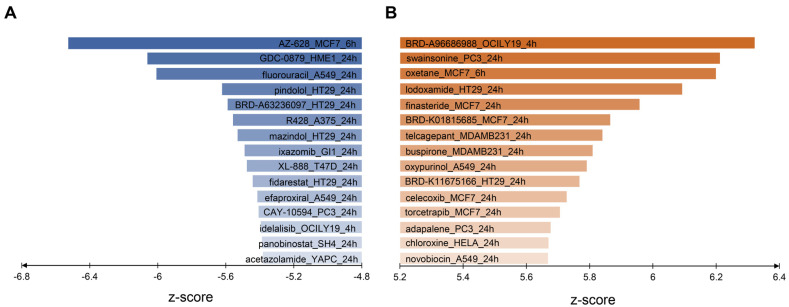
The predicted m6A-targeted drug candidate through Connectivity Map (CMap) analysis. (**A**) Reverser drugs obtained by CMap analysis of consensus DEGs. Each bar label shows the corresponding drug name/code, treated cell line and treatment time. (**B**) Mimicker drugs obtained by CMap analysis of consensus DEGs. Each bar label shows the corresponding drug name/code, treated cell line and treatment time.

We performed a CMap analysis to identify drugs based on the consensus DEGs. We first filtered the consensus DEGs to produce a strong consensus m6A-related DEG signature, and analysis was performed on these filtered consensus DEGs. Two categories of drugs could be predicted in this comparative analysis. The first category is the reversers, which have the reversed direction of differential expression after drug treatment compared to with the differential expression of consensus genes. Intuitively, the reversers are predicted to reduce the m6A methylation. The second category are the mimickers, which have a similar direction of differential expression after drug treatment compared to the differential expression of consensus genes. Presumably, these mimickers may also elevate the cellular level of m6A methylation. Notably, in order to find drugs most likely related to cancers, we only retained the studies with doses of less than 2 μmol while using cancer cell lines. After the screening, we obtained top reverser and mimicker drugs from the CMap analysis (Figure 2). Interestingly, known drugs with the anti-cancer activity such as fluorouracil [22], idelalisib [23] and Panobinostat [24] are among the top of this list. Additionally, AZ628_MCF7 and R428_A375 were selected from the reversers for further experiments to verify whether they could indeed inhibit cancer cell proliferation and reduce m6A methylation.

### 3.4. AZ628 Inhibits MCF7 Cell Proliferation and Reduces Cellular m6A Methylaiton

**Figure 3:**
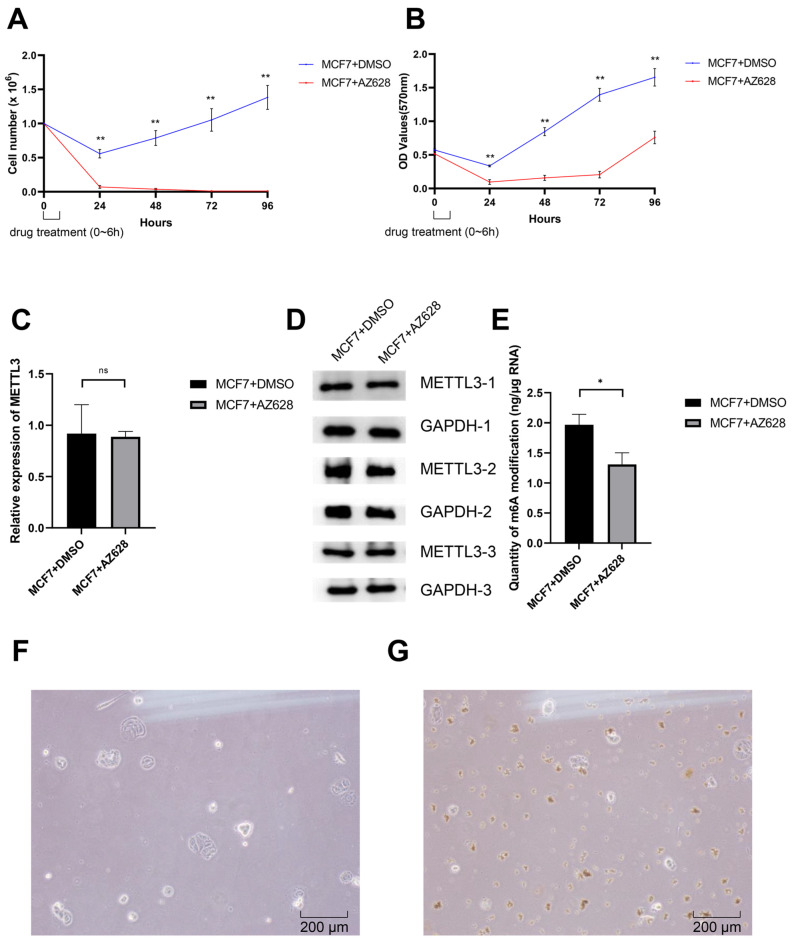
Effect of AZ628 on human breast cancer cell line MCF7. (**A**,**B**) The proliferation of human breast cancer cell line MCF7 was significantly inhibited by AZ628 treatment. The results from the cell count assay (**A**) and the MTT assay (**B**) are shown. Data represented the mean ± SEM from five independent experiments, *t*-test, ** *p* < 0.01. Some SEM values in the drug-treated experimental group that are too small to be shown are specified here: (**A**) 24 h. 0.021; 48 h, 0.013; 72 h, 0.001; 96 h, 0.001; (**B**) 24 h. 0.035; 48 h, 0.038; 72 h, 0.047; 96 h, 0.094. (**C**,**D**) The protein expression of METTL3 in the MCF7 cell line was not significantly affected by drug treatment, *t*-test, none significance (ns). The summary of Western blot grey values as the mean ± SEM from three independent experiments (**C**) and the representative Western blot result (**D**) are shown. (**E**) The RNA m6A modification level in the MCF7 cell line was significantly decreased after AZ628 treatment. Data represented the mean ± SEM from five independent experiments, *t*-test, * *p* < 0.05. (**F**,**G**) Representative images of treated and control cells observed under an inverted 10× microscopy.

According to the results obtained by our screening, AZ628 has the strongest correlation with the genes we screened; therefore, we chose to use AZ628 in the treatment of the human breast cancer cell line MCF7 to evaluate the possible effect of this drug. The CMap analysis indicates that MCF7 treated with AZ628 for 6 h show the strongest reversal signature to the consensus m6A-related DEG signature (Figure 2A); therefore, we also selected 6 h as the treatment time for AZ628. After 6 h of treatment, the cells in the culture dishes were digested and counted at 24, 48, 72 and 96 h to observe the possible effect of AZ628 on cell proliferation. Indeed, the number of cells in the experimental group decreased significantly after the treatment of the DMSO-dissolved AZ628, and gradually approached zero. The number of cells in the control group decreased only within 24 h after DMSO solute treatment, and the cell proliferation was then recovered (Figure 3A). To further validate the results from the cell count assay, we checked the cell viability after drug treatment using the MTT cell proliferation and cytotoxicity assay kit (Figure 3B). In the results of the MTT assay, the experimental group also exhibited prominent cell proliferation inhibition compared to the control group. To further investigate whether the inhibition was related to m6A modification, a Western blot was used to observe whether the expression of METTL3 in the m6A writer was affected. The results show that there was no significant difference in METTL3 expression between the experimental group and the control group after drug treatment (Figure 3C,D). However, the amount of m6A modification in the experimental group is significantly reduced, which indicates that AZ628 can inhibit m6A methylation (Figure 3E). Direct observation of the cells from the control group (Figure 3F) and the treated group (Figure 3G) under 10× inverted microscope reveals that many cells in the experimental group would undergo apoptosis, in which cell contents are spilled, and cell proliferation is affected.

### 3.5. R428 Inhibits A375 Cell Proliferation and Reduces Cellular m6A Methylaiton

**Figure 4:**
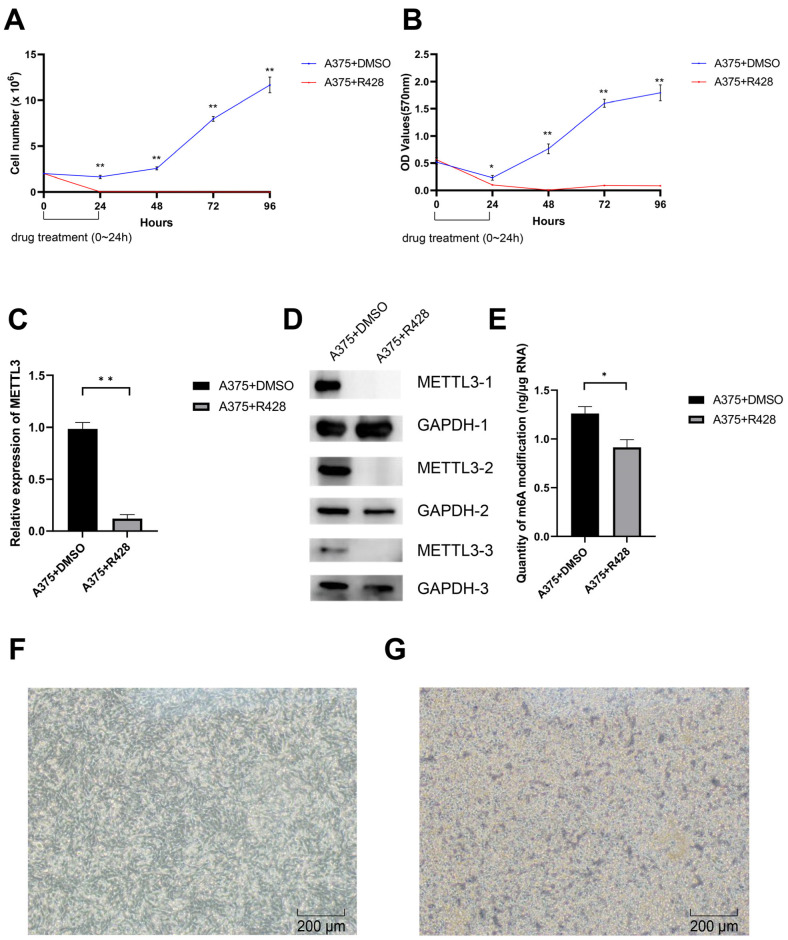
Effect of R428 on human melanoma cell line cell line A375. (**A**,**B**) The proliferation of the human breast cancer cell line A375 was significantly inhibited by R428 treatment. The results from the cell count assay (**A**) and the MTT assay (**B**) are shown. Data represented the mean ± SEM from five independent experiments, *t*-test, * *p* < 0.05, ** *p* < 0.01. Some SEM values in the drug-treated experimental group that are too small to be shown are specified here: (**A**) 24 h. 0.009; 48 h, 0.012; 72 h, 0.006; 96 h, 0.000; (B) 24 h. 0.004; 48 h, 0.002; 72 h, 0.006; 96 h, 0.002. (**C**,**D**) The protein expression of METTL3 in the A375 cell line was significantly reduced by drug treatment, *t*-test, ** *p* < 0.01. The summary of Western blot grey values as the mean ± SEM from three independent experiments (**C**) and the representative Western blot result (**D**) are shown. (**E**) The RNA m6A modification level in the A375 cell line was significantly decreased after R428 treatment. Data represented the mean ± SEM from five independent experiments, *t*-test, * *p* < 0.05. (**F**,**G**) Representative images of treated and control cells observed under an inverted 10× microscopy.

Based on our screening results and available laboratory materials, we chose to treat the human melanoma cell line A375 with R428 to evaluate the possible effect of this drug. The CMap analysis indicates that A375 treated with R428 for 24 h show the strongest reversal signature to the consensus m6A-related DEG signature (Figure 2A); therefore, we also selected 24 h as the treatment time for R428. After 24 h of treatment, the cells in the culture dishes were digested and counted at 24, 48, 72 and 96 h to observe the possible effect of AZ628 on cell proliferation. Compared with the control group, the number of A375 cells in the experimental group decreased to nearly zero at 24 h after treatment (Figure 4A). The MTT assay also verified the prominent cell growth inhibition effect of R428 on the A375 cells (Figure 4B). The results of the Western blot show that the expression of the m6A writer METTL3 could hardly be observed in the A375 cells of the experimental group (Figure 4C,D), and the amount of m6A modification in the experimental group was also significantly decreased (Figure 4E), suggesting that R428 may play an anticancer effect by inhibiting m6A modification. Under the inverted 10× microscope, we found that the cells in the experimental group (Figure 4F) after drug treatment show an apoptotic phenotype. Compared to the normal cells (Figure 4G) the lysosomes lysed the cells and formed holes in the cells.

## 4. Discussion

Considering the extensive regulatory effects of m6A methylation in the onset and progression of cancers, the m6A methylation pathway has become an intriguing target for novel anti-cancer therapies. As with the results, new drug candidates that have potential to interfere with the m6A pathway are of particular interest. The Connectivity Map (CMap) is a widely acknowledged resource for drug repurposing and drug discovery [25]. Conceptually, this is also applicable for screening drugs that inhibit cancer cell proliferation through regulating the cellular m6A level. However, since diverged DEGs can be obtained in different datasets with different cancer type backgrounds and different m6A-related perturbations, a pipeline to identify consensus m6A-related DEG signature was first established. The results also confirm that CMap analysis results based on the consensus m6A-related DEGs covered several known drugs that were reported to have anti-cancer potential. For example, meta-analysis shows that Fluorouracil, a drug predicted by our analysis, significantly reduced mortality, metastasis, and recurrence rates in colorectal cancer patients [22]. Another predicted drug, Fluorouracil, had anticancer effects by binding to RNA and promoting disruption of RNA synthesis [26]. In addition, idelalisib, a PI3K inhibitor, may unleash antitumor T-cell responses by inhibiting regulatory T cells and immune-suppressive myeloid cells [23]. Finally, panobinostat was used clinically to treat multiple myeloma [24], and several studies demonstrate its anti-cancer effect [27,28,29].

For the drug predictions verified in this study, R428 is an inhibitor of Axl, a receptor tyrosine kinase (RTK) which is widely distributed in epithelial, mesenchymal and hematopoietic cell lines [30,31]. Axl can interact with the growth arrest specific protein 6 and promote cell proliferation but suppress apoptosis [32]. R428 plays a significant anticancer effect by inhibiting Axl; however, according to our study, the m6A writer METTL3 and m6A methylation may also contribute to the effect of R428. We observed a significant reduction of cellular m6A level as well as the protein expression of the m6A writer METTL3, which also provides a new way to explain the anticancer effect of R428. AZ628 can bind to the substrate site of ATP-Binding Cassette Transporter G2 (ABCG2) and then affect ABCG2-mediated anticancer drug efflux, thereby exerting anticancer effects [33]. In our study, we found that the anti-cancer effect of AZ628 may also be related to m6A modification. Although no significant inhibition of METTL3 expression is observed, a significant reduction of m6A modification is still observed in drug-treated cells.

Our computational analysis provides new candidate m6A-targeted drugs that inhibit cancer cell proliferation. However, as an observational study, one limitation of the current analysis is that it does not confirm the exact targeting mechanism of these drugs. The direct targeting mechanism of these drugs would be tested by biophysical techniques, or the indirect targeting mechanism would be further interpreted by combing further ad hoc omics data and molecular biology experiments. In the future, further exploration of the role of m6A in cancer can provide new ideas for the development of anticancer drugs and help to find more drugs with potential anticancer effects.

## Figures and Tables

**Table 1:** Transcriptome datasets with m6A perturbation covered in this study.

GEO ID	Perturbation	Cancer Type
GSE147884	*METTL3* KD	prostate cancer
GSE161302	*METTL3* KD	prostate cancer
GSE161301	*METTL3* KD	esophageal cancer
GSE182382	*METTL3*/*ALKBH5* KD	colorectal cancer
GSE190078	*METTL3* KD	pancreatic cancer
GSE95372	*METTL3* KO	cervical cancer
GSE130012	*METTL3* KO	colorectal cancer
GSE179267	*METTL3* overexpression	esophageal cancer
GSE81164	*METTL14* KD	breast cancer
GSE186581	*METTL14* overexpression	oral squamous cell carcinoma
GSE103497	*FTO* KD	acute monocytic leukemia
GSE107411	*FTO* KD	liver cancer
GSE133517	*FTO* KD	cervical cancer
GSE128574	*ALKBH5* KD	monocytic leukemia
GSE144959	*ALKBH5* KD	acute myeloid leukemia
GSE144968	*ALKBH5* KD	acute myeloid leukemia
GSE146874	*ALKBH5* KD	myeloid leukemia

## Data Availability

Publicly available datasets were analyzed in this study. This data can be found in Gene Expression Omnibus (GEO) database (https://www.ncbi.nlm.nih.gov/geo/), with the accessions of GSE81164, GSE95372, GSE103497, GSE107411, GSE128574, GSE130012, GSE133517, GSE144959, GSE144968, GSE146874, GSE147884, GSE161301, GSE161302, GSE179267, GSE182382, GSE186581, and GSE190078.
